# Tumor Necrosis Factor Superfamily 14 (LIGHT) Restricts Neovascularization by Decreasing Circulating Endothelial Progenitor Cells and Function

**DOI:** 10.3390/ijms24086997

**Published:** 2023-04-10

**Authors:** Chien-Yi Hsu, Chun-Yao Huang, Chun-Ming Shih, Yi-Wen Lin, Po-Hsun Huang, Shing-Jong Lin, Chen-Wei Liu, Cheng-Yen Lin, Feng-Yen Lin

**Affiliations:** 1 Taipei Heart Institute and Division of Cardiology, Department of Internal Medicine, Taipei Medical University, Taipei 110, Taiwan; 2 Division of Cardiology, Department of Internal Medicine, Taipei Medical University Hospital, Taipei 110, Taiwan; 3 Department of Biomedical Sciences and Engineering, National Central University, Taoyuan City 320, Taiwan; 4 Institute of Oral Biology, National Yang Ming Chiao Tung University, Taipei 112, Taiwan; 5 Division of Cardiology, Taipei Veterans General Hospital, Taipei 112, Taiwan; 6 Department of Basic Medical Science, College of Medicine, University of Arizona, Phoenix, AZ 85721, USA; 7 Healthcare Information and Management Department, Ming Chuan University, Taoyuan 333, Taiwan

**Keywords:** tumor necrosis factor superfamily 14, LIGHT, endothelial progenitor cells

## Abstract

Tumor necrosis factor superfamily 14 (TNFSF14) is also known as the LT-related inducible ligand (LIGHT). It can bind to the herpesvirus invasion mediator and lymphotoxin-β receptor to perform its biological activity. LIGHT has multiple physiological functions, including strengthening the synthesis of nitric oxide, reactive oxygen species, and cytokines. LIGHT also stimulates angiogenesis in tumors and induces the synthesis of high endothelial venules; degrades the extracellular matrix in thoracic aortic dissection, and induces the expression of interleukin-8, cyclooxygenase-2, and cell adhesion molecules in endothelial cells. While LIGHT induces tissue inflammation, its effects on angiogenesis after tissue ischemia are unclear. Thus, we analyzed these effects in the current study. In this study, the animal model of hind limb ischemia surgery in C57BL/6 mice was performed. Doppler ultrasound, immunohistochemical staining, and Western blotting were employed to analyze the situation of angiogenesis. In addition, human endothelial progenitor cells (EPCs) were used for in vitro studies to analyze the possible mechanisms. The results in the animal study showed that LIGHT injection inhibited angiogenesis in ischemic limbs. For the in vitro studies, LIGHT inhibited the expression of integrins and E-selectin; decreased migration and tube formation capabilities, mitochondrial respiration, and succinate dehydrogenase activity; and promoted senescence in EPCs. Western blotting revealed that the impairment of EPC function by LIGHT may be due to its effects on the proper functioning of the intracellular Akt signaling pathway, endothelial nitrite oxide synthase (eNOS), and mitochondrial respiration. In conclusion, LIGHT inhibits angiogenesis after tissue ischemia. This may be related to the clamped EPC function.

## 1. Introduction

Impaired angiogenesis and decreased capillary density are one of the causes of coronary artery disease (CAD) [1]. Patients with cardiovascular disease often have high concentrations of the blood tumor necrosis factor (TNF). TNF is an inflammatory cytokine that is mainly synthesized by monocytes and macrophages, which increases the expression of adhesion molecules and the scavenger receptor in monocytes. This increased expression promotes the formation of foam cells, plaques, and thrombi and increases the risk of vascular endothelial dysfunction, atherosclerosis, and CAD [2,3]. Additionally, the tumor necrosis factor (TNF) superfamily is a group of proteins that share structural similarities with TNF. Interactions between members of the TNF superfamily (TNFSF) and TNF receptor superfamily (TNFRSF) activate the nuclear factor-kappa B (NF-κB) signaling pathway; promotes inflammation; and also regulates cell survival, proliferation, and differentiation [4]. TNFSF14 is also known as the LT-related inducible ligand that competes for glycoprotein D binding to the herpesvirus entry mediator on T cells (LIGHT). Additionally, it can bind to the herpesvirus invasion mediator and lymphotoxin-β receptor to perform its biological activity [5,6]. Previous reports demonstrated that LIGHT signaling strengthens the synthesis of nitric oxide (NO), reactive oxygen species, and cytokines and maintains the stability of lymphoid organs, liver, and bones [7,8]. LIGHT also induces angiogenesis and endothelization in tumors [9]. During the development of thoracic aortic dissection, LIGHT is important in extracellular matrix degradation [10]. LIGHT interacts with soluble TNFRSF6B/decoy receptor 3 (DcR3, a soluble receptor with neutralizing effects on LIGHT) in endothelial cells. The subsequent activation induces the expression of interleukin-8 (IL-8), cyclooxygenase-2 (COX-2), and cell adhesion molecules (CAMs), which promote vascular inflammation, atherosclerosis [11], and vascular restenosis after angioplasty intervention [12].

The endothelium is a monolayer structure made up of endothelial cells (ECs) that is in direct contact with the blood. The endothelium can secrete NO, heparin, plasmin, and other regulatory factors to inhibit coagulation and maintain vascular patency [13,14]. When the endothelium is dysfunctional, it will lead to cardiovascular diseases. Injured blood vessels and ischemic tissues need to restore their function and perfusion by reendothelization and neovascularization/angiogenesis, respectively, in which EPCs play an important role. During the process of reendothelialization and neovascularization/angiogenesis, CD34^+^/CD133^+^/kinase insert domain receptor (KDR)^+^/VEGF receptor 2 (VEGFR2)^+^, and expressing endothelial progenitor cells (EPCs) migrate from the bone marrow to the peripheral blood. After EPCs have migrated to the target site, neovascularization/angiogenesis and reendothelialization will take place through the capacities of migration, proliferation, and differentiation of EPCs [15,16,17]. In fact, the function of EPCs profoundly affects the recovery state of vascular function after injury. Previous researchers have also evidenced that a decrease in the number of circulating EPCs is positively correlated with cardiovascular events, and increased numbers of EPCs may reverse vascular endothelial dysfunction [18,19]. Bacterial endotoxins, exotoxins, inflammatory cytokines, and reactive oxygen species (ROS) can affect the number of circulating EPCs and the differentiation and function of EPCs [20]. Therefore, the strategies of anti-inflammation and antioxidation may be helpful to the biological function of EPCs and reverse the decline.

The function of EPC is to determine the conditions of reendothelialization and neovascularization/angiogenesis after vascular injury. LIGHT may participate in various hyperinflammatory responses and pathological processes caused by arterial endothelial injury [21,22]. Even so, there is still a lack of understanding of the mechanism by which LIGHT affects post-ischemic angiogenesis. Therefore, in this study, we examined the effects of LIGHT on EPC function after tissue hypoxia and observed its effects on angiogenesis.

## 2. Results

### 2.1. LIGHT Decreases the Recovery of Capillary Density in C57BL/6 Mice

**Figure 1:**
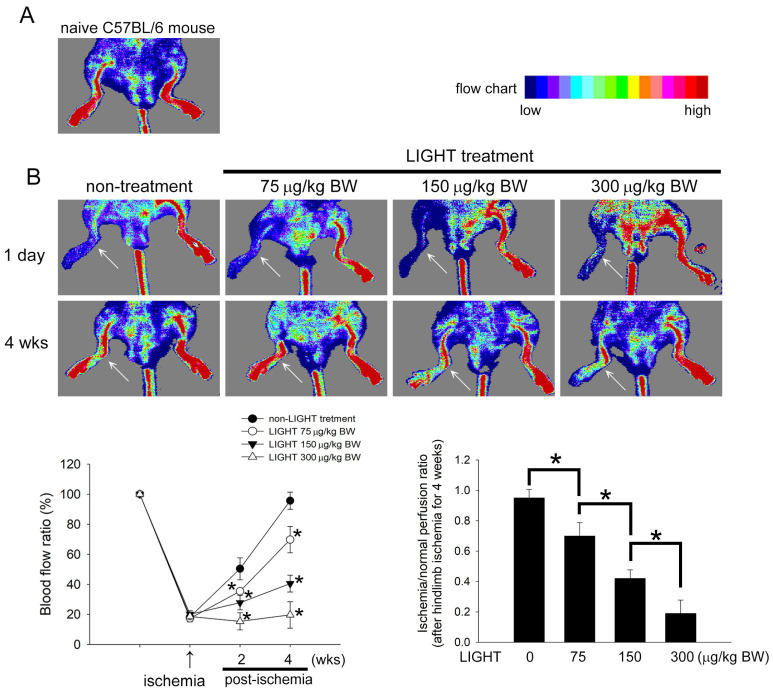

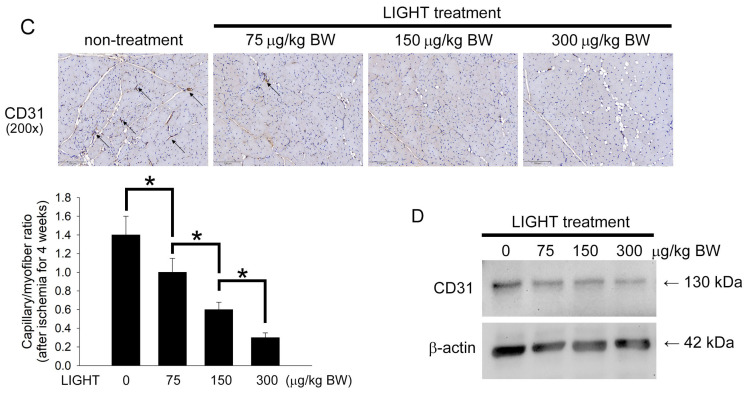
LIGHT decreases the recovery of capillary density in hind limb ischemia C57BL/6 mice. (**A**) Representative preoperative laser Doppler hind limb blood flow results in mice. (**B**) The upper panel shows representative lower limb blood flow results using laser Doppler measurements at 1 day and 4 weeks after hind limb ischemia surgery in mice from various groups. The color scale shows blood flow from lowest to highest values. The white arrow indicates the ischemic limb after hind limb ischemia surgery. The lower left panel shows changes in blood flow ratio (ischemia/non-ischemia) with time in various groups after surgery. Results are expressed as the mean ± standard deviation (SD). * *p* < 0.05 was considered statistically significant compared to the non-LIGHT treatment group at the same time point. The lower right panel presents a bar graph. The data demonstrate the significantly lower ischemia/normal perfusion ratio of the LIGHT treatment groups compared to that of the nontreatment group 4 weeks after hind limb ischemia surgery, regardless of whether 75, 150, or 300 μg/kg BW LIGHT was injected. (**C**) Immunohistochemical staining was performed 4 weeks after surgery to observe the vascular endothelial cell marker expressing cells in muscle tissues below the ligated blood vessel. The black arrow shows CD31 expression in the blood vessel. Tissues are presented at 200× magnification in the light microscopy image. The bar graph shows the statistical results of capillary density (capillary/myofiber ratio) for each group of animals (*n* = 5). Results are expressed as the mean ± SD. * *p* < 0.05 was considered significant. (**D**) Representative Western blotting results show the CD31 level in muscle tissues extracted 4 weeks after surgery in mice.

To evaluate the effects of LIGHT on angiogenesis, a C57BL/6 mouse model of ice unilateral hind limb femoral artery ligation was used. The laser Doppler analysis revealed that bilateral femoral arteries in mice were filled with blood (Figure 1A). One day after ligation and resection of the right femoral artery, the blood flow was effectively blocked (Figure 1B, upper column). Laser Doppler analysis at 4 weeks after ligation showed that the blood flow in the ischemic limb was restored to nontreatment levels. However, mice given LIGHT showed a significant delay in blood flow recovery after ischemia, and blood flow recovery was most impeded in mice injected with 300 μg/kg BW LIGHT. Blood flow was restored to 95.7 ± 5.7% 4 weeks after hind limb ischemia surgery (left lower image in Figure 1B). Blood flow recovery after treatment with 75, 150, and 300 μg/kg BW LIGHT was 69.8 ± 8.8%, 40.5 ± 5.6%, and 19.7 ± 8.5%, respectively. The ischemia/normal perfusion ratios of the LIGHT treatment groups were significantly decreased compared with those of the nontreatment group at 4 weeks after hind limb ischemia surgery (right lower image in Figure 1B). The immunohistochemical assay was used to analyze the microvascular expression in perfused tissues after ligation. Immunohistochemical staining with CD31 as a marker showed that LIGHT treatment inhibited angiogenesis after hind limb ischemia surgery (Figure 1C). The bar graph clearly showed that LIGHT significantly decreased the capillary/myofiber ratio in tissue sections. Lastly, Western blotting analysis of the vascular content using the tissue CD31 expression level revealed that CD31 expression was inhibited due to LIGHT treatment. These results prove that LIGHT inhibits capillary density recovery after hind limb ischemia in C57BL/6 mice.

### 2.2. LIGHT Decreases the Number of Circulating EPCs In Vivo and Inhibits EPCs Differentiation Ex Vivo

**Figure 2:**
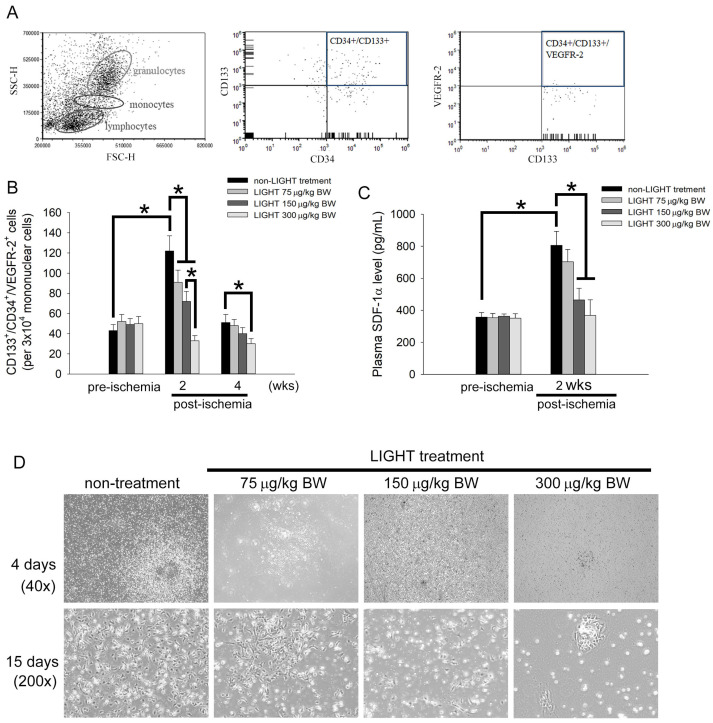

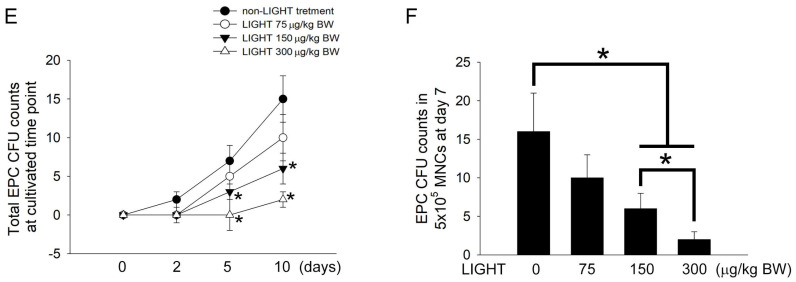
LIGHT inhibits the mobilization and differentiation of circulating EPCs in hind limb ischemia C57BL/6 mice. (**A**) Flow cytometry analysis of the levels of circulating EPCs in C57BL/6 mice. Circulating EPCs are defined as CD133^+^/CD34^+^/VEGFR-2^+^ cells. (**B**) Level of CD133^+^/CD34^+^/VEGFR-2^+^ cells in untreated and LIGHT-treated C57BL/6 mice before surgery and 2 and 4 weeks after surgery. (**C**) ELISA data of the blood SDF-1α concentration in mice before and 2 weeks after surgery. (**D**) At the end of the experiment (4 weeks), mice were sacrificed, and MNCs were extracted and cultured in EGM-2 culture medium. From day 2 of the culture onward, the cells were observed using microscopy once every day. Magnified images (200×) of cell cultures at days 4–15 are shown. The arrows show EPC colony formation. (**E**) EPC colony formation on days 2, 5, and 10 of cell cultures. The unit is colony-forming units. (**F**) Bar graph of the number of EPC colonies formed on day 7 of the culture (5 × 10^5^ MNCs/per well in a 6-well plate). All results are expressed as the mean ± standard deviation (*n* = 5). * *p* < 0.05 was considered significant.

The number of circulating EPCs and EPC differentiation are key to angiogenesis. Flow cytometry and the colony-forming assay were used to observe the effects of LIGHT on EPC mobilization in C57BL/6 mice. In flow cytometry, circulating EPCs were defined as CD133^+^/CD34^+^/VEGFR-2^+^ cells in peripheral blood (Figure 2A). No change in the baseline EPC value in blood was evident before the experiment in all the groups (Figure 2B). The number of circulating EPCs in C57BL/6 mice was significantly increased 2 weeks after surgery compared with that before hind limb ischemia surgery. However, the number of circulating EPCs was significantly deceased in C57BL/6 mice that were treated with LIGHT compared with that in the nontreated mice, and the magnitude of the decrease was positively correlated with the LIGHT dose. The number of circulating EPCs recovered to near baseline levels in the different groups 4 weeks after hind limb ischemia surgery. Stromal cell-derived factor 1 alpha (SDF-1α) is a main factor driving bone marrow EPC mobilization. Therefore, we used ELISA to analyze the plasma SDF-1α level 2 weeks after surgery. No difference in the plasma SDF-1α levels was evident between the different groups of mice before the experiment (Figure 2C). Hind limb ischemia surgery caused the plasma SDF-1α level to increase. C57BL/6 mice treated with LIGHT had significantly decreased SDF-1α production after hind limb ischemia, especially LIGHT injected with 150 and 300 μg/kg BW. Figure 2D shows the capability of C57BL/6 mouse MNCs to differentiate into early EPC colonies. After culturing for 4 days, MNCs from the non-LIGHT treatment group grew into colonies with a central core of spherical cells. The ability of MNCs from LIGHT-treated mice to differentiate into early EPC colonies was inhibited. When MNCs were cultured for 15 days, almost all MNCs in the non-LIGHT treatment group have completely differentiated into fusiform or cobblestone-like EPCs. However, the LIGHT treatment groups showed sparse cell distribution; even MNCs from the 150 and 300 μg/kg BW treatment groups only differentiated into a few early EPC colonies. Figure 2E shows the number of EPC colonies on days 2, 5, and 10 of the MNC cultures in the different groups. Figure 2F shows the number of EPC colonies on day 7 of the MNC cultures in animals in different groups. These results showed that LIGHT will inhibit the mobilization and differentiation of circulating EPCs after hind limb ischemia surgery.

### 2.3. LIGHT Induces Senescence and Mitochondrial Dysfunction in Human EPCs

**Figure 3:**
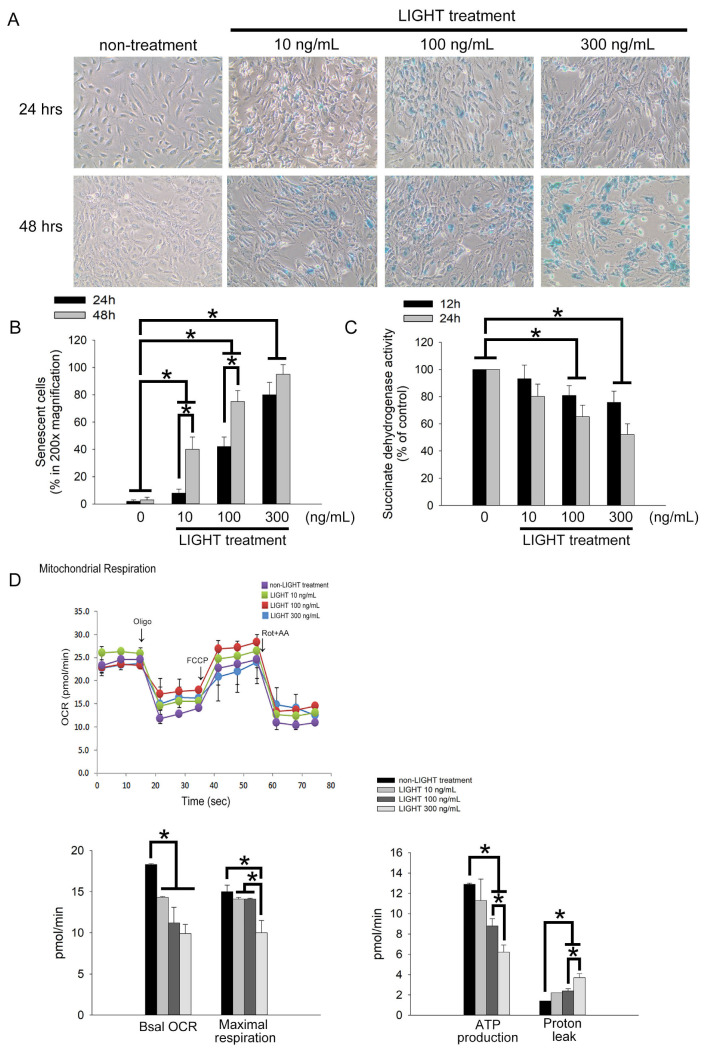
LIGHT stimulates human EPC senescence by destroying mitochondria function and succinate dehydrogenase (SDH) activity. (**A**) EPCs were treated with 10, 100, or 300 ng/mL recombinant LIGHT for 24 or 48 h. Cell senescence was analyzed; the photographs present representative results. Senescent EPCs display accumulated blue β-galactosidase in 100× magnification. (**B**) Quantification of senescent EPCs. (**C**) EPCs were treated with 10, 100, or 300 ng/mL recombinant LIGHT for 12 or 24 h. The intracellular activity of SDH was analyzed. (**D**) The Seahorse XFp platform was used to analyze the changes in the cell oxygen consumption rate (OCR) with time for different experimental groups (pmol/min). The arrows show the time when oligomycin (oligo), FCCP, or rotenone+antimycin (Rot+AA) was added. Four replicate wells were used for each time point. Basal OCR, maximal respiration, ATP production, and proton leak were calculated based on the Seahorse XFp analysis theory and software. Three independent experiments were carried out in every group, and different cell generations were used for every independent experiment. All results are expressed as the mean ± standard deviation (*n* = 5). * *p* < 0.05 was considered significant.

Senescence limits the replicative capability of normal cells and causes cells to be maintained in a viable but senescent state. Senescence is a characteristic of poor EPC function. The Senescence Cell Staining kit was used to evaluate the effects of LIGHT on EPC senescence. EPCs were treated with 10–300 ng/mL LIGHT, and a senescence analysis was performed 24 or 48 h after treatment. Senescent cells with blue crystals increased after LIGHT treatment (Figure 3A). These cells were enumerated. The results in Figure 3B show that, compared with that in the non-LIGHT treatment group, the percentage of senescence-associated β-galactosidase-positive EPCs was significantly increased in the LIGHT treatment (10 ng/mL LIGHT in the 48 h group: 40.0 ± 9.3% of the control, 100 ng/mL LIGHT in the 24 and 48 h groups: 42.6 ± 7.3% and 75.6 ± 8.3% of the control, respectively, and 300 ng/mL LIGHT in the 24 and 48 h groups: 80.0 ± 9.3% and 95.6 ± 8.3% of the control, respectively). The main cause of cell senescence is decreased intracellular SDH activity. Therefore, we used SDH as a marker to analyze the effects of LIGHT on EPCs. Compared with that in the non-LIGHT treatment group, the percentage of SDH activity in EPCs was significantly inhibited by the LIGHT treatment (100 ng/mL LIGHT in the 12 and 24 h groups: 80.9 ± 7.2% and 65.3 ± 8.5% of the control, respectively, and 300 ng/mL LIGHT in the 12 and 24 h groups: 75.8 ± 8.3% and 52.1 ± 7.9% of the control, respectively) (Figure 3C). Mitochondrial function affects the biological activity of cells. During senescence, mitochondrial respiration and energy production are decreased. Therefore, we used the Seahorse XFp platform to analyze the effects of LIGHT on mitochondrial function in EPCs. Figure 3D shows the changes in the oxygen consumption rate (OCR) in EPCs with time. During the analyze process, oligo, FCCP, and Rot+AA were successively added, and Seahorse XFp analysis software was used to calculate the basal OCR, maximal respiration, ATP production, and proton leakage. LIGHT treatments of 100 and 300 ng/mL consistently decreased the basal OCR, maximal respiration, and ATP production and increased the proton leakage in EPC mitochondria (Figure 3E,F). These results show that LIGHT disrupts mitochondrial function and SDH activity to cause senescence in EPCs.

### 2.4. LIGHT Decreases Neovasculogenesis of Human EPCs

**Figure 4:**
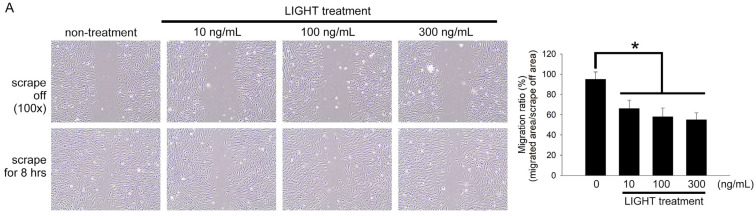

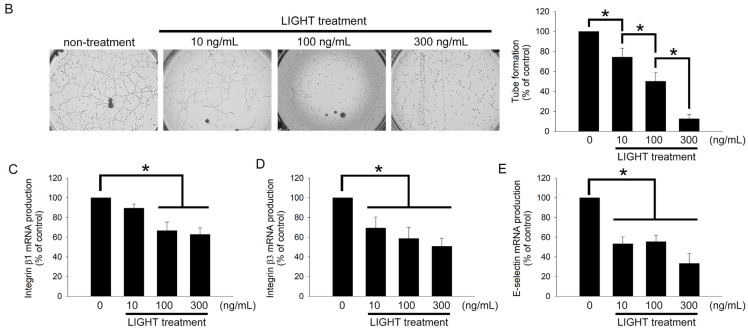
LIGHT reduces the migration and tube-forming capabilities in human EPCs by reducing the expression of adhesion molecules. (**A**) Migration assay was used to analyze the effects of LIGHT on EPC migration. The upper row shows the scratches (dotted lines). The lower row shows cell migration 8 h after scratching. Migration ratio (migrated area/scrape off area) was calculated using 100× magnification. (**B**) An angiogenesis assay kit was used to analyze the tube-forming capability results of EPCs. The numbers in three tubes in every group were calculated. The results are presented in the right figure. (**C**–**E**) Real-time PCR was used to analyze integrin β1, integrin β3, and E-selectin mRNA expression in EPCs from the different experimental groups. All data are expressed as the mean ± standard deviation of three independent experiments. Statistical evaluations were performed using the Student’s *t*-test, followed by Dunnett’s test. * *p* < 0.05 was considered significant.

Migration and tube formation capabilities are the basic functions of EPC in angiogenesis. These functions can be used as markers of EPC capability. EPCs were treated with 10, 100, or 300 ng/mL LIGHT for 24 h before a micropipette yellow tip was used to make a scratch on the cells growing on the surface of a culture dish. EPCs in the non-LIGHT treatment group almost covered the scratch 8 h after scratching (migrated area/scrape off area = 95.0 ± 4.2%; Figure 4A). Compared with that in the non-LIGHT treatment group, the migration capability of EPCs was inhibited after LIGHT treatment. Compared with that in the non-LIGHT treatment group, the migration ratio was significantly decreased with LIGHT treatment (10 ng/mL LIGHT treatment group: 66.0 ± 8.4%, 100 ng/mL LIGHT treatment group: 58.6 ± 8.7%, and 300 ng/mL LIGHT treatment group: 55.0 ± 6.8%) (Figure 4B). Similarly, the tube-forming capability of EPCs was decreased due to LIGHT treatment. Compared with that in the non-LIGHT treatment (control) group, the tube formation ratios in the 10, 100, and 300 ng/mL LIGHT treatment groups were 74.2 ± 9.1%, 50.1 ± 8.8%, and 12.5 ± 4.2% of the control, respectively (Figure 4B). Integrin β1, integrin β3, and selectin are important adhesion molecules for the homing, migration, and tube formation of EPCs. We employed real-time PCR to measure the effects of LIGHT on EPCs. LIGHT decreased the expression of adhesion molecules in EPCs (Figure 4C–E). The collective results show that LIGHT decreases the expression of adhesion molecules to decrease the migration and tube-forming capabilities in EPCs.

### 2.5. Akt- and eNOS-Related Pathways May Contribute to the Functional Disruption of Human EPCs by LIGHT

**Figure 5:**
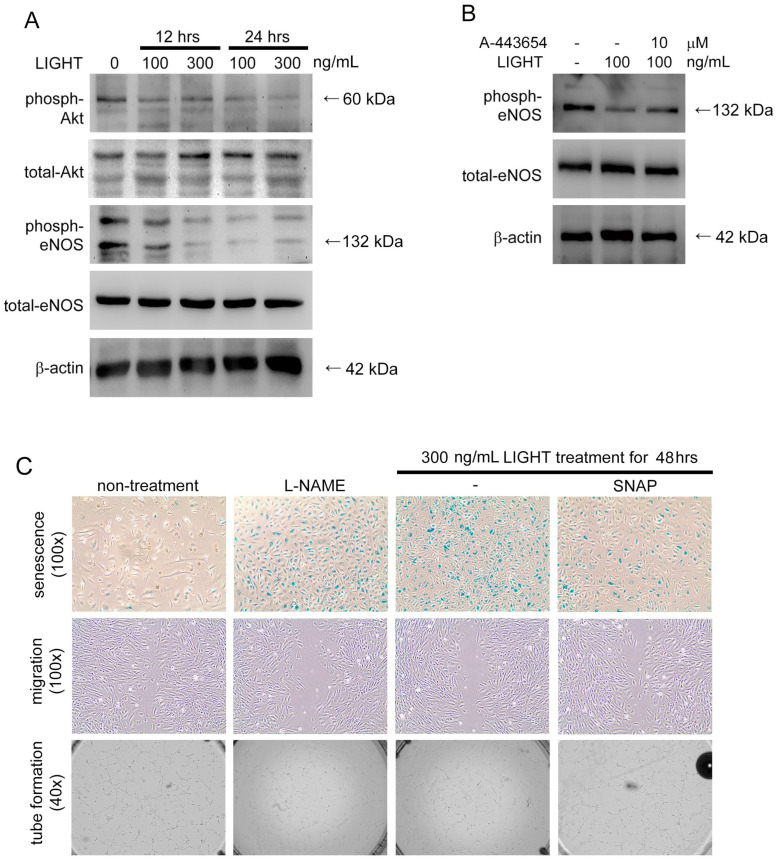

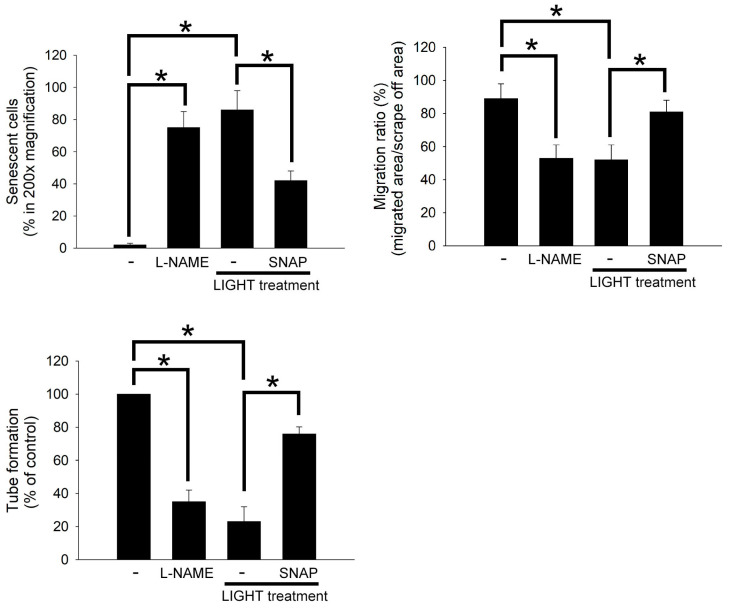
Senescence and dysfunction caused by LIGHT in human EPCs may be via the Akt-eNOS axis pathway. (**A**) LIGHT was used to stimulate human EPCs for 12 or 24 h before the total protein was extracted for Western blotting. (**B**) Akt inhibitor A-443654 was used to treat human EPCs for 6 h, followed by 300 ng/mL LIGHT treatment for 24 h. Total protein was extracted for Western blotting. Total-Akt and β-actin expression levels were used as control for the amount of protein loaded. (**C**) L-NAME (10 μM) was used to treat human EPCs for 48 h, or LIGHT was used to treat human EPCs for 48 h without or with a 1-h pretreatment with 10 μM SNAP. Data on senescence, migration, and tube formation capabilities in human EPCs are presented. The lower three figures show the senescence, migration, and tube formation quantitation results. All data are expressed as the mean ± standard deviation of three independent experiments. Statistical evaluations were performed using the Student’s *t*-test, followed by Dunnett’s test. * *p* < 0.05 was considered significant.

Previous studies showed that the Akt-eNOS axis pathway regulates neovasculogenic function in EPCs. We performed a Western blot analysis to assess if the effects of LIGHT on EPCs are due to the regulation of the Akt-eNOS axis pathway. LIGHT inhibited the phosphorylation of Akt and activation of eNOS in EPCs (Figure 5A). Akt inhibitor A-443654 treatment reduced the suppression of eNOS activity in LIGHT-stimulated EPC (Figure 5B). L-N^G^-Nitro arginine methyl ester (L-NAME; a non-selective NOS inhibitor) significantly inhibited EPC senescence and migration and tube formation capabilities in EPCs (Figure 5C). The pretreatment of EPCs with S-nitroso-N-acetylpenicillamine (SNAP; a NO donor) could improve senescence and inhibition of migration and tube formation caused by LIGHT. These results show that the senescence and inhibition of neovasculogenesis in EPCs caused by LIGHT may be through the Akt-eNOS axis pathway.

## 3. Discussion

In this study, it was observed that LIGHT decreased angiogenesis in C57BL/6 mice after hind limb ischemia surgery. In vitro human EPC experiments demonstrated that LIGHT increases senescence and inhibits migration and tube formation and that these effects increase as the LIGHT dose increases. This may be the reason for decreased angiogenesis after hind limb ischemia surgery. To the best of our knowledge, this is the first study to report that LIGHT has negative effects on late EPCs. In addition, LIGHT can regulate the vasculogenic ability of EPCs through Akt- and NOS-mediated mechanisms.

There has been no consistent and authoritative result on the correlation between blood LIGHT concentration and cardiovascular events. Our recent published results found that stable CAD patients with elevated plasma LIGHT [23] and DcR3 [24] have a high risk of cardiovascular events after percutaneous coronary intervention (PCI) treatment. Some researchers believe that increased circulating high-sensitivity C-reactive protein (hsCRP) levels in CAD patients after PCI can be a marker of systemic inflammatory status [25]. However, the accuracy and predictive power of hsCRP remain contentious [26,27,28,29]. In one of our previous studies, we found that the addition of LIGHT significantly increased the predictive power of hsCRP concerning total severe cardiovascular events [23]. We believe that LIGHT has an additional predictive power for the risk of cardiovascular events in cardiovascular disease patients. Based on the results of our and other studies, we believe that the combined use of LIGHT and determination of the hsCRP levels can more effectively and accurately predict the risk of redeveloping cardiovascular events in stable CAD patients who have undergone PCI.

Previously, the plasma LIGHT level in CAD patients was reported to be significantly higher than that in non-CAD subjects [30]. Therefore, an elevated circulating LIGHT level may be associated with acute myocardial infarction, ischemic stroke, and other cardiovascular diseases. The role of LIGHT in atherosclerosis is still unconfirmed. The LIGHT level in circulating blood has been positively correlated with the N-terminal-pro hormone brain natriuretic peptide (NT-pro-BNP), adiponectin, TNF-α, and matrix metalloproteinase-9 (MMP-9) levels [23]. These are also factors that are intimately associated with atherosclerosis. In ECs, the interaction between LIGHT and decoy receptor 3 (DcR3; which can be considered to be an immune modulator) affects inflammatory responses in cells. Therefore, circulating LIGHT may affect the vascular and cardiac inflammation status and can be used as a marker for evaluating systemic inflammatory responses. The occurrence of cardiovascular disease is associated with immune and inflammatory responses. LIGHT participates in innate and adaptive immune responses and is associated with many autoimmune diseases [5]. In vascular tissues undergoing atherogenic or inflammatory responses, platelets, activated/infiltrating T cells, and monocytes that adhere to the vascular endothelium are important sources of LIGHT [31,32]. These low amounts of secreted LIGHT induce inflammatory responses in vascular ECs, including the activation of protease-activated receptor 2 on ECs [21]. Furthermore, circulating LIGHT does not seem to originate from vascular or infiltrating/activated cells. However, the activity of hepatic lipoprotein lipase in the liver will decrease due to the effects of circulating LIGHT, causing lipid accumulation and hypertriglyceridemia [33]. Under hyperlipidemic conditions, LIGHT also causes the uptake of modified lipids to increase in macrophages [30]. In summary, LIGHT induces inflammatory responses in vascular cells. However, the specific mechanism by which it induces atherosclerosis and cardiovascular events have not been completely elucidated.

In addition to immune responses, LIGHT also affects mitochondrial respiration and function. Similar to TNF-α, LIGHT also belongs to the TNF superfamily. LIGHT can promote the activation and proliferation of T cells to increase interferon-gamma (IFN-γ) secretion, further increasing the cytotoxicity of T lymphocytes in many autoimmune diseases [34,35]. The binding of LIGHT and IFN-γ will activate NF-κB and increase mitochondrial permeabilization, further inducing apoptosis [36].

In addition to binding to the lymphotoxin-β receptor (LTβR) and herpesvirus entry mediator (HVEM), LIGHT can also bind to proteins [37]. The interactions between these different ligands and receptors are complex. For example, LTβR binds to lymphotoxin αβ heterotrimers expressed on cell surfaces [38]. A recent animal experiment in a mouse model of colitis found that the binding of LIGHT and LTβR limited glycolysis, respiration, and reactive oxygen species production in the mitochondria of neutrophils, and the inhibition of LTβR expression reversed severe colitis [39]. Liver mitochondria controls hepatocyte energy metabolism through ATP synthesis and fatty acid oxidation [40]. In TNFSF14 knockout mice, LIGHT loss increases liver lipolysis [41] and mitochondrial respiratory changes [42]. These findings echo the present findings that LIGHT decreased the aerobic metabolic rate and the amount of ATP synthesized in the mitochondria of EPCs. This causes mitochondrial dysfunction and senescence. Thus, LIGHT can affect intracellular mechanisms.

In conclusion, it was demonstrated in this study that LIGHT reduces post-ischemic angiogenesis by negatively affecting the function of EPCs, although we only presented some of the possible mechanisms. In the future, exploring and understanding the molecular mechanisms can effectively maintain the function of EPCs in the process of inflammation.

## 4. Materials and Methods

### 4.1. Reagents

LIGHT was purchased from ProSpec-Tany TechnoGene Ltd. (Ness-Ziona, Israel).

### 4.2. Animal Management and Grouping

Male C57BL/6 mice were purchased from BioLASCO, I-Lan Taiwan Co., Ltd. The source of the JAX^®^ 003752 mice was The Jackson Laboratory (Bar Harbor, ME, USA). All animals were handled according to protocols approved by the Institutional Animal Care Committee of Taipei Medical University (LAC-2018-0249). The experimental procedures and animal care conformed to the “Guide for the Care and Use of Laboratory Animals” (NIH Publication No. 85-23, revised 1996) published by the United States National Institutes of Health. All mice were reared in microisolator cages with alternating 12-h periods of light and dark and were given ad libitum access to food (Scientific Diet Services, Essex, UK). The 12 C57BL/6 mice were divided into four groups. The control group underwent only hind limb ischemia surgery. The three IS+LIGHT treatment groups underwent ischemia surgery, followed by tail vein injection of 75, 150, or 300 μg/kg body weight (BW) of LIGHT daily from day 2 onwards. The animals were sacrificed 4 weeks after surgery.

### 4.3. Hind Limb Ischemia Surgery and Doppler Analysis

Six-week-old C57BL/6 mice weighing 20–25 g were selected. After immobilization, xylocaine (2 mg/kg BW) plus Zoletil (5 mg/kg BW; containing a dissociative anesthetic, tiletamine/zolazepam, at a ratio of 1:1) was administered by intraperitoneal injection for anesthesia. First, the lower abdomen was shaved. An 8 mm incision was made at the connection between the abdomen and right lower limb. The femoral artery was located using microscopy, and the nerves and veins were dissected. Ligation was performed at the front end of the femoral artery and the rear end 5 mm away. The blood vessel between the two ligation sites was excised before suturing the skin to close the opening to prevent subsequent infection. A Doppler imaging system (Moor Instruments Limited, Devon, UK) was used for measurement while avoiding the effects of ambient temperature and lighting as much as possible. The blood flow in mice was recorded. Analysis was performed 1 day, 2 weeks, and 4 weeks after the surgery. The ratio of the perfusion between the ischemic right limb and the non-ischemic left limb was estimated. At the end of the 4-week experiment, the animals were anesthetized before they were euthanized by cervical dislocation.

### 4.4. Immunohistochemical Staining

After the animals were euthanized, the muscles below the ligated blood vessels (sartorius, gracilis, adductor, and semimembranosus muscles) were collected and fixed by immersion in 4% paraformaldehyde (Sigma-Aldrich, St. Louis, MO, USA). Paraffin (Sigma-Aldrich, St. Louis, MO, USA) embedding was performed, followed by sectioning of the tissue into 5-μm-thick sections. Rabbit anti-CD31 antibody (Millipore, Billerica, MA, USA) was used for the immunohistochemical staining of ischemic thigh muscles in mice. Microscopy was used to view three tissue cross-sections from each animal, and 10 different fields were selected for each tissue. The number of capillaries seen was calculated. The number of capillaries was expressed as a capillary/myofiber ratio.

### 4.5. Western Blot Analysis

Protein lysis buffer was added to homogenized muscle tissues or EPCs for lysis to extract protein. SDS-PAGE was used to separate proteins with different molecular weights before their transfer to a PVDF membrane. Anti-CD31, anti-β-actin, anti-eNOS, anti-phosphorylated eNOS (Millipore, Temecula, CA, USA), anti-Akt, and anti-phosphorylated Akt (Cell Signaling Technology, Danvers, MA, USA) antibodies were used for detection. ECL (Amersham Biosciences, Piscataway, NJ, USA) was used for the chemiluminescence reaction, and ImageQuant LAS 400 (GE Healthcare, Chicago, IL, USA) was used to measure the protein blots.

### 4.6. Flow Cytometry

Two hundred to four hundred microliters of RBC lysis buffer (BD Biosciences, Santa Clara, CA, USA) were added to one hundred to two hundred microliters of peripheral blood. This was followed by two washes with PBS to isolate the total leukocytes. Cy5-conjugated anti-mouse CD34 (eBioscience, San Diego, CA, USA), Alexa 488-conjugated anti-mouse VEGFR-2 (Flk-1, eBioscience, San Diego, CA, USA), and phycoerythrin (PE)-conjugated anti-mouse CD133 antibodies (eBioscience, San Diego, CA, USA) were added to the total leukocytes and incubated for 1 h. A flow cytometer (Becton Dickinson, San Jose, CA, USA) was used to measure the EPC content in the samples. Each analysis was performed on ~15,000–30,000 total leukocytes. Circulating EPCs were considered to be derived from the monocyte population and confirmed by triple positivity for CD34, CD133, and VEGFR-2.

### 4.7. Mouse Early EPCs Colony-Forming Assay

During animal sacrifice, cardiac puncture was used to obtain 2 mL of blood from the heart. Histopaque-1083 (Sigma-Aldrich, St. Louis, MO, USA) was used to isolate the total mononuclear cells (MNCs). The obtained MNCs (5 × 105) were inoculated onto 2 mL of endothelial growth medium-2 (EGM-2; Clonetics Lonza Ltd., Alpharetta, GA, USA) and cultured in a 37 °C, 5% CO_2_ incubator. After 7 days of culture, the culture medium was carefully replaced to remove nonadherent cells. Early EPCs remained adhered to the culture dish. The culture medium was changed every 3 days, and daily observation and enumeration were used to monitor the number of colonies.

### 4.8. Isolation and Culture of Human EPCs

Forty milliliters of venous blood donated by volunteers underwent density centrifugation using Histopaque-1077 (Sigma-Aldrich, St. Louis, MO, USA) to isolate the MNCs. The cells were mixed with EGM-2, and complete culture medium with supplements was added. The cells were then cultured at 37 °C. After 4 days, nonadherent cells were removed, and the culture medium was changed. The medium was changed every 3 days thereafter. The number of colonies was observed and calculated. Early EPCs appeared as long spindles. After 2–4 weeks, some cells grew into cobblestone-like late EPCs.

### 4.9. EPCs Senescent Assay

The Senescence Cell Staining kit (Sigma-Aldrich, St. Louis, MO, USA) was used to evaluate the EPC senescence according to the manufacturer’s instructions. We added 5 × 10^4^ EPCs to the 24-well culture plate in each hole and treated them with LIGHT. β-Galactosidase-positive senescent cells appeared blue. The relative percentage of blue cells to total cells was calculated to evaluate the senescence level of the EPCs.

### 4.10. Succinate Dehydrogenase (SDH) Activity

3-(4,5-Dimethylthiazol-2-yl)-2,5-diphenyltetrazolium bromide (MTT; Sigma-Aldrich, St. Louis, MO, USA) was used to analyze the effects of LIGHT on SDH activity in EPCs. EPCs were seeded in 96-well plates containing 10, 100, or 300 ng/mL LIGHT and cultured for 12 or 24 h, followed by the addition of MTT. After 4 h, dimethyl sulfoxide was used to dissolve intracellular crystals, and the absorbance was measured at 550/650 nm.

### 4.11. Bioenergetic Analysis

ATP synthesis represents biological energy. The Seahorse XFp platform (Agilent, San Diego, CA, USA) was used to measure the mitochondrial oxidative phosphorylation and oxygen consumption in the surrounding environment of cells to determine the energy metabolism status of the cells. One day before loading, a fluorescent probe was activated in the calibration solution. Two hundred microliters of correction solution were added to every well in the analysis plate. The pH of the correction solution was first adjusted to 7.4 to avoid affecting the experiment results. The solution was stored at 37 °C in a CO_2_-free environment before use. Four hundred microliters of sterile water were added to the eight grooves around the cell and probe plates to maintain the temperature and humidity. EPCs (104) were added to every well in the cell plate. The EPCs were treated with LIGHT at 37 °C for 24 h. One hour before loading, the cell culture growth medium was replaced with 180 μL of serum free-medium containing 2 mM L-glutamine to prevent other substances in the culture medium from affecting the experiment results. Twenty microliters of 0.5 mM Oligomycin (Oligo), twenty-two microliters 0.5 mM carbonyl cyanide-p-trifluoromethoxyphenylhydrazone (FCCP), and twenty-five microliters of 0.25 mM Rotenone/Antimycin A (Rot+AA) were added to the A, B, and C grooves in the probe plate, respectively. The aerobic metabolic rate and mitochondrial stress of the cells were measured. Finally, the Bradford assay was used to measure the corrected protein concentrations of the cells in the cell plate, followed by the WAVE software analysis.

### 4.12. EPC Migration Assay

The migration of late EPCs is important to angiogenesis. This assay was used to evaluate the effects of LIGHT on the EPC migration capability. Human EPCs were seeded in 12-well plates and treated with LIGHT for 24 h. A 200 μL genomic pipette tip (Cat. No.: T118RS-Q, Thermo^®^ OSP, CA, USA) was used to make a 300 μm scratch. After 8 h, the scratch was examined by optical microscopy using a model IX71 instrument (Olympus, Tokyo, Japan) and photographed with an attached Macro FIRE 2.3A CCD camera. Six random microscopy fields were used to calculate the degree of EPC migration in the scratch after treatment with different LIGHT concentrations.

### 4.13. EPC Tube Formation Assay

Late EPC tube formation is important for angiogenesis. An Angiogenesis Assay Kit (Chemicon, Temecula, CA, USA) was used to evaluate the effects of LIGHT on EPC tube formation. Thawed ECMatrix gel solution was mixed with diluent and added to 96-well plates. The plates were incubated at 37 °C for 1 h for solidification. EPCs treated with LIGHT for 24 h were collected, and 10^3^ cells were added to the solidified matrix. The cells were cultured at 37 °C for 12 h. An inverted optical microscope was used for observation. Four fields were randomly selected for photography in each well. The lumen-like structures with intact peripheries were calculated and compared the mean values of four field in each hole in EPCs treated with different concentrations of LIGHT.

### 4.14. Real-Time Polymerase Chain Reaction (PCR)

Extracted total RNA was used for reverse transcription and quantitative real-time PCR. The PCR primers for integrin 1, integrin 2, and E-selectin are shown in Table 1. Glyceraldehyde 3-phosphate dehydrogenase (GAPDH) mRNA was used as an internal control.

### 4.15. Statistical Analyses

Values are expressed as the mean ± standard deviation. One-way analysis of variance and post hoc tests were used for the statistical analysis, followed by Tukey’s test. A *p*-value < 0.05 was considered statistically significant.

## 5. Conclusions

LIGHT inhibits angiogenesis after tissue ischemia. The LIGHT inhibition of the intracellular Akt-eNOS signaling axis and mitochondrial respiration in EPCs impairs cell function. These events are the possible causes of impaired angiogenesis.

## Figures and Tables

**Table 1:** PCR primers used for the polymerase chain reactions.

Gene	Sequence
integrin β1	forward: 5′-ctg gtg tgg ttg ctg gaa ttg ttc-3′
	reverse: 5′-cct cat act tcg gat tga cca cag-3′
integrin β3	forward: 5′-cct gct cat ctg gaa act cct ca-3′
	reverse: 5′-cgg tac gtg ata ttg gtg aag gta g-3′
E-selectin	forward: 5′-ttg gta gct gga ctt tct gct gc-3′
	reverse: 5′-gta aga agg ctt ttg gta gct tcc-3′
GAPDH	forward: 5′-tgc ccc ctc tgc tga tgc c-3′
	reverse: 5′-cct ccg acg cct gct tca cca c-3′

GAPDH, glyceraldehyde 3-phosphate dehydrogenase.

## Data Availability

Not applicable.
